# Supplementary material for: STING mediates immune responses in the closest living relatives of animals
Source: eLife. 2021 Nov 3;10:e70436. doi: 10.7554/eLife.70436 (PMC8592570; doi:10.7554/eLife.70436)

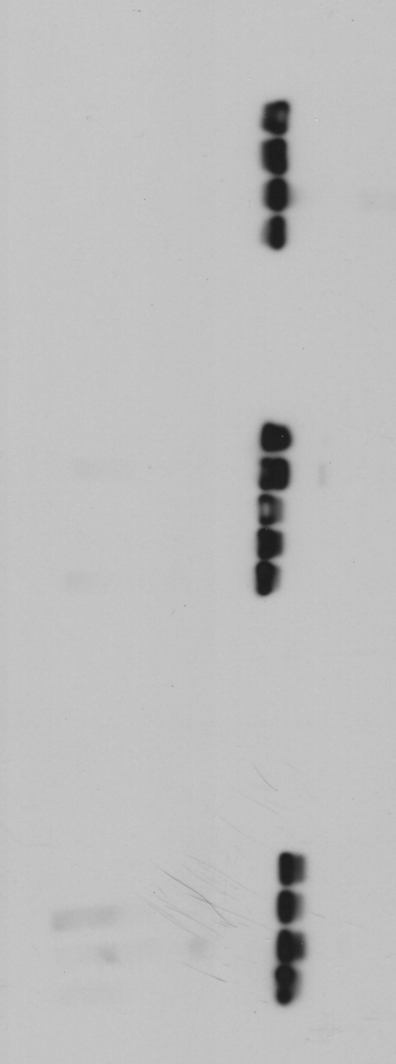

Supplement: Source data 1. [file elife-70436-supp2.zip › Western Blots Source Data/Fig2C+3E_Tub.pdf]
