# Supplementary figures and images for: STING mediates immune responses in the closest living relatives of animals
Source: eLife. 2021 Nov 3;10:e70436. doi: 10.7554/eLife.70436 (PMC8592570; doi:10.7554/eLife.70436)

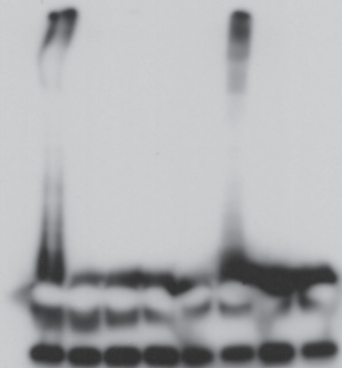

Fig. 1. Gel electrophoresis of DNA.

Supplement: Source data 1. [file elife-70436-supp2.zip › Western Blots Source Data/Fig6B_Tub.pdf]

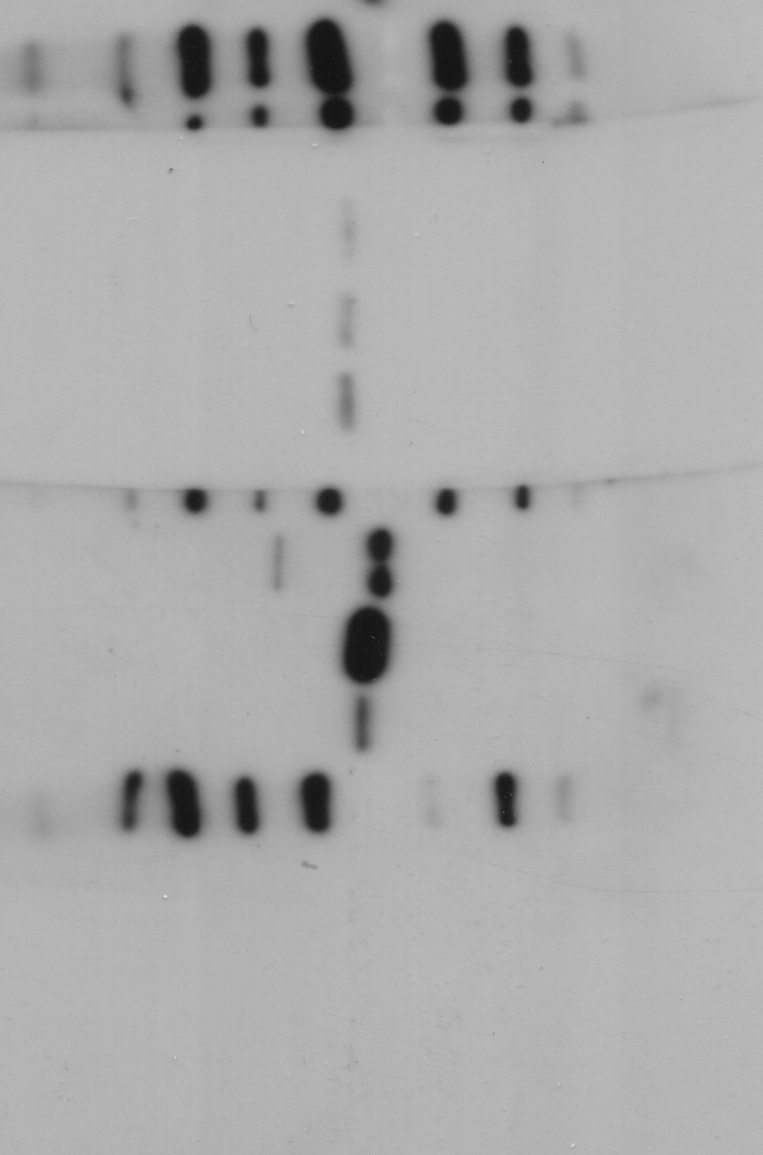

Supplement: Source data 1. [file elife-70436-supp2.zip › Western Blots Source Data/Fig5B_Tub+STING.pdf]

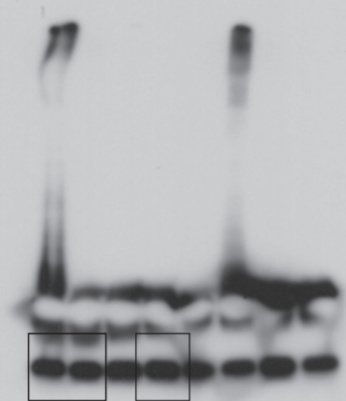

Supplement: Source data 1. [file elife-70436-supp2.zip › Western Blots Source Data/Fig6B_Tub_highlighted.pdf]

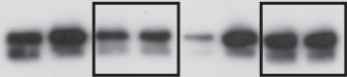

Supplement: Source data 1. [file elife-70436-supp2.zip › Western Blots Source Data/Fig6_S1B_Tub_highlighted.pdf]

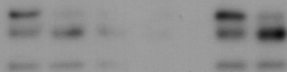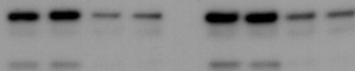

Supplement: Source data 1. [file elife-70436-supp2.zip › Western Blots Source Data/Fig6A_mCherry.pdf]

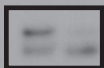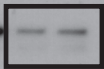

Supplement: Source data 1. [file elife-70436-supp2.zip › Western Blots Source Data/Fig6A_mCherry_highlighted.pdf]

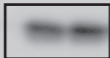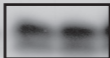

Supplement: Source data 1. [file elife-70436-supp2.zip › Western Blots Source Data/Fig6A_Tub_highlighted.pdf]

Tubulin

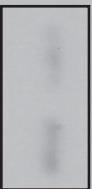

STING

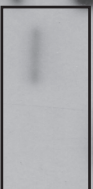

Supplement: Source data 1. [file elife-70436-supp2.zip › Western Blots Source Data/Fig5B_Tub+STING+highlighted.pdf]

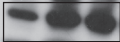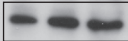

Supplement: Source data 1. [file elife-70436-supp2.zip › Western Blots Source Data/Fig2C_STING_highlighted.pdf]

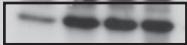

Supplement: Source data 1. [file elife-70436-supp2.zip › Western Blots Source Data/Fig3E_STING_highlighted.pdf]

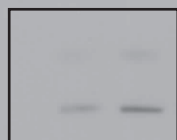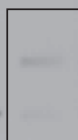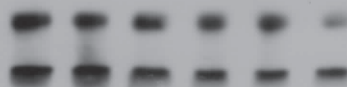

Supplement: Source data 1. [file elife-70436-supp2.zip › Western Blots Source Data/Fig6B_mCherry_highlighted.pdf]

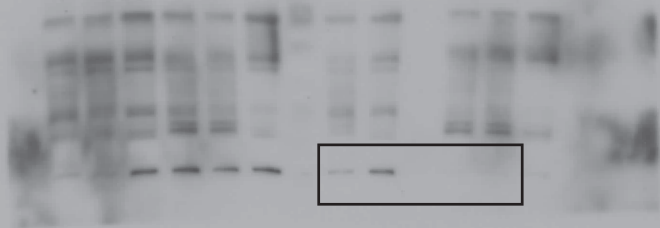

Supplement: Source data 1. [file elife-70436-supp2.zip › Western Blots Source Data/Fig6A_STING_highlighted.pdf]

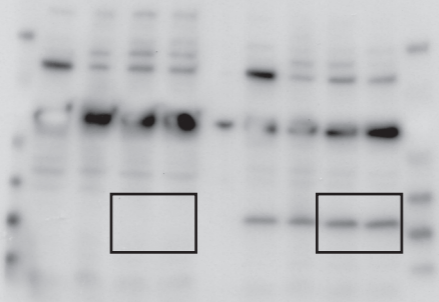

Supplement: Source data 1. [file elife-70436-supp2.zip › Western Blots Source Data/Fig6_S1B_STING_highlighted.pdf]

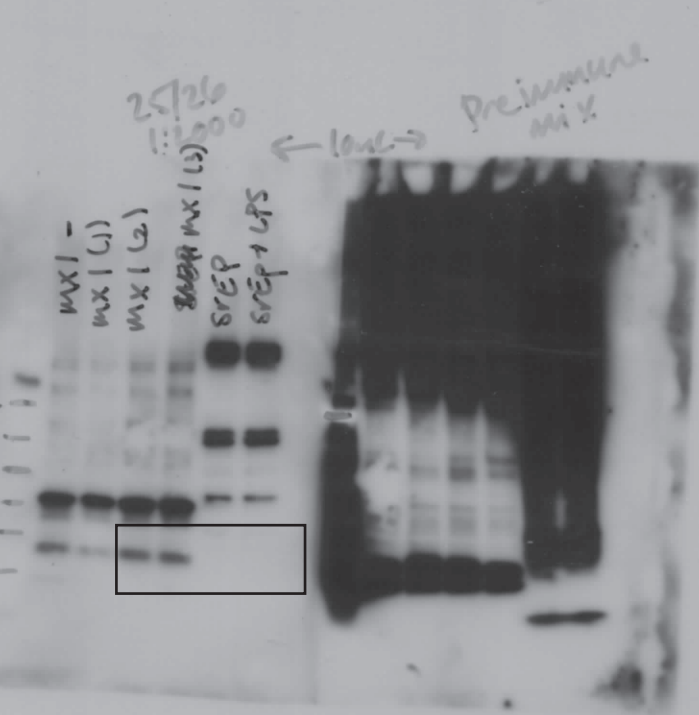

Boiled samples x 5 min  
@ 100°C

(1) → puffer LPS  
(2) → winter LPS  
(3) → mirisogen LPS } for

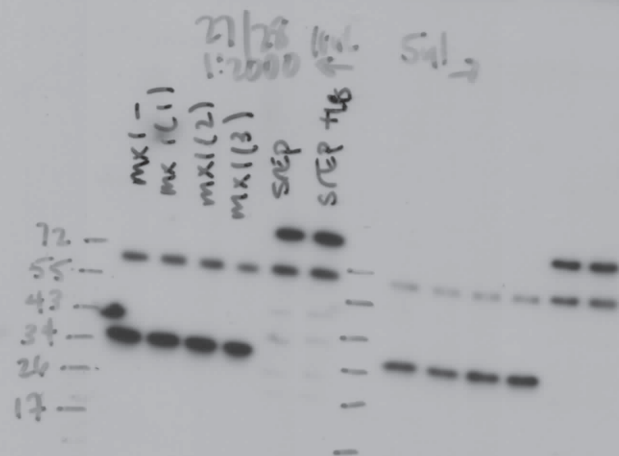

Supplement: Source data 1. [file elife-70436-supp2.zip › Western Blots Source Data/Fig2_S1C_STING_highlighted.pdf]

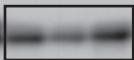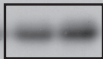

Supplement: Source data 1. [file elife-70436-supp2.zip › Western Blots Source Data/Fig3D_tubulin_highlighted.pdf]

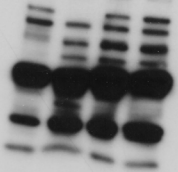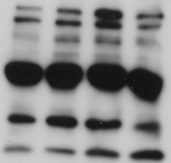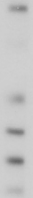

Supplement: Source data 1. [file elife-70436-supp2.zip › Western Blots Source Data/Fig2C_STING.pdf]

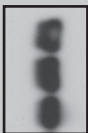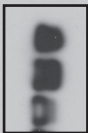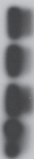

Supplement: Source data 1. [file elife-70436-supp2.zip › Western Blots Source Data/Fig2C_Tub_highlighted.pdf]

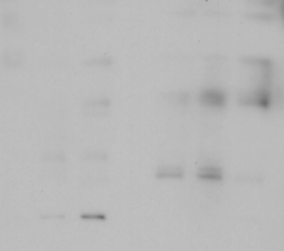

Supplement: Source data 1. [file elife-70436-supp2.zip › Western Blots Source Data/Fig6A_STING.pdf]

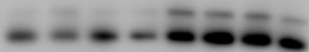

Supplement: Source data 1. [file elife-70436-supp2.zip › Western Blots Source Data/Fig6_S1B_mcherry.pdf]

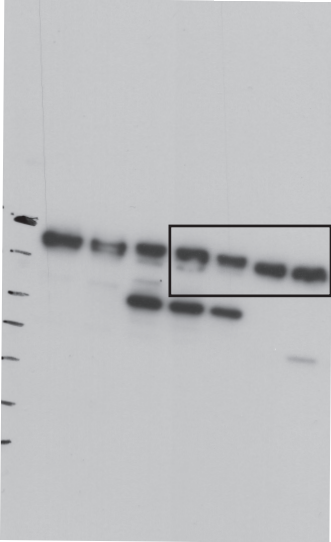

Supplement: Source data 1. [file elife-70436-supp2.zip › Western Blots Source Data/Fig2_S1C_Tubulin_highlighted.pdf]

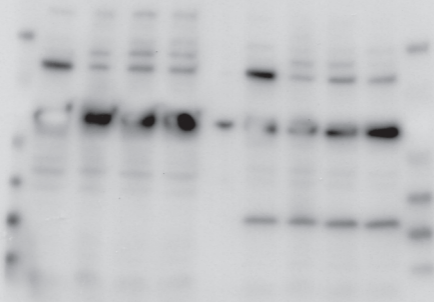

Supplement: Source data 1. [file elife-70436-supp2.zip › Western Blots Source Data/Fig6_S1B_STING.pdf]

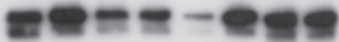

Supplement: Source data 1. [file elife-70436-supp2.zip › Western Blots Source Data/Fig6_S1B_Tub.pdf]

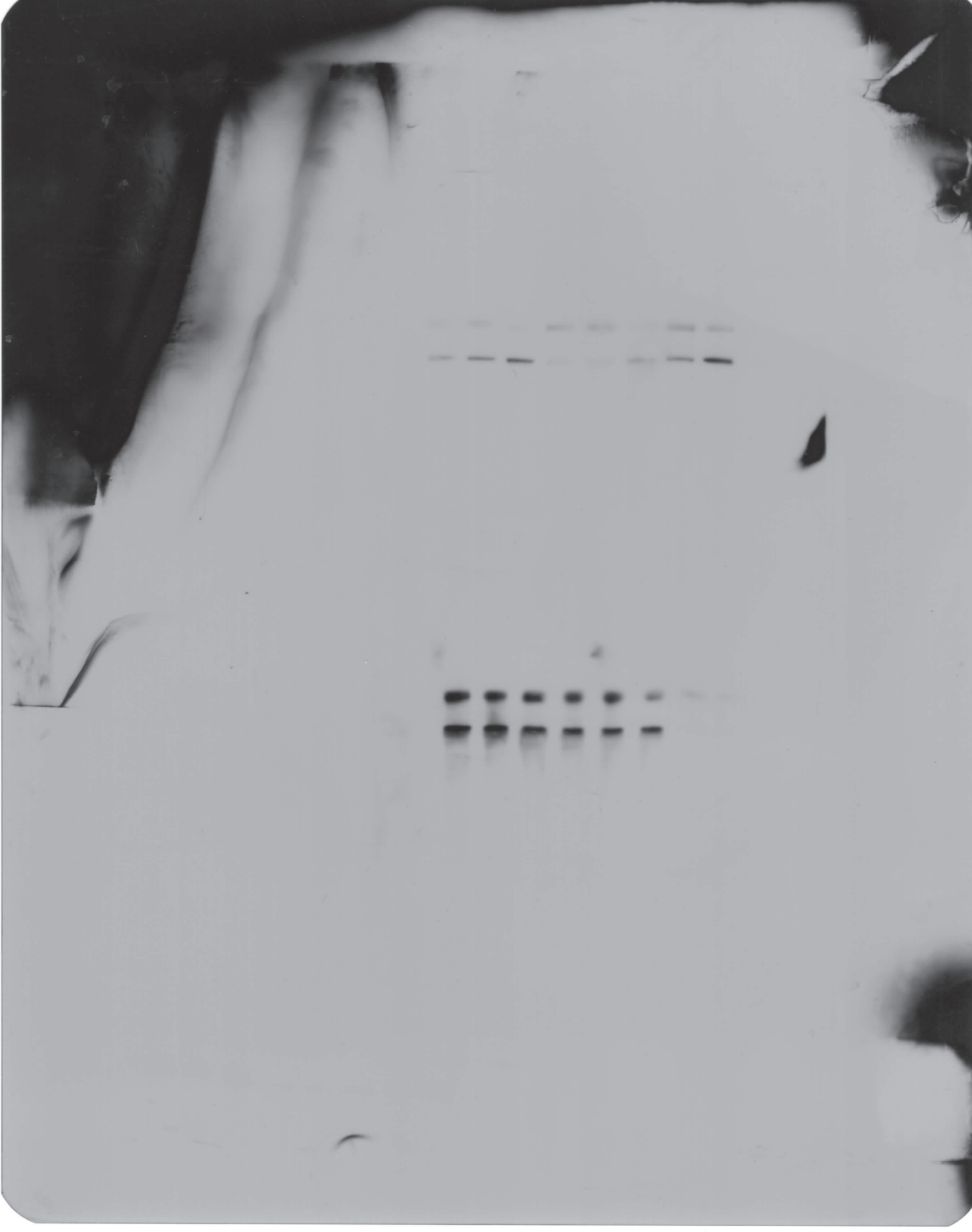

Supplement: Source data 1. [file elife-70436-supp2.zip › Western Blots Source Data/Fig6B_mCherry_uncropped.pdf]

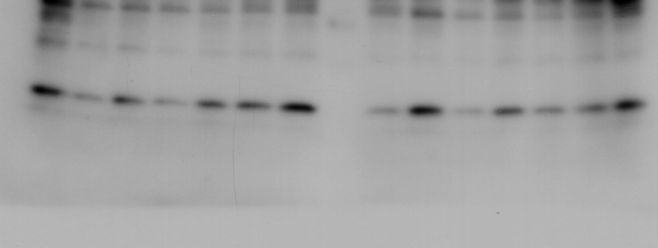

Supplement: Source data 1. [file elife-70436-supp2.zip › Western Blots Source Data/Fig3D_STING.pdf]

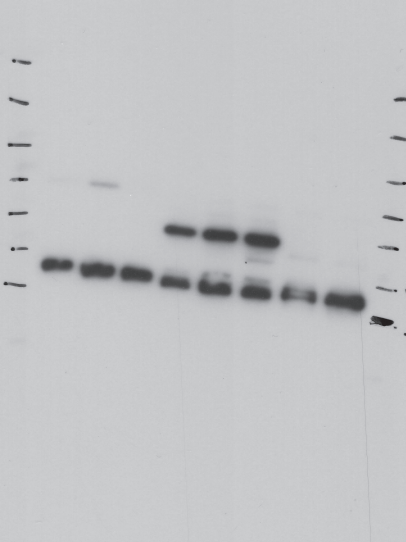

Supplement: Source data 1. [file elife-70436-supp2.zip › Western Blots Source Data/Fig2_S1C_tubulin_uncropped.pdf]

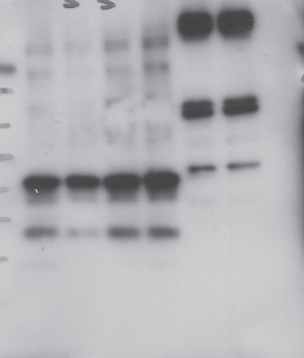

Supplement: Source data 1. [file elife-70436-supp2.zip › Western Blots Source Data/Fig2_S1C_STING.pdf]

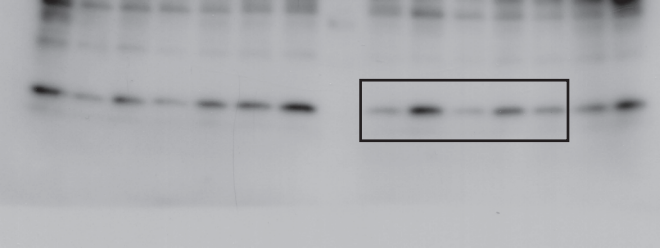

Supplement: Source data 1. [file elife-70436-supp2.zip › Western Blots Source Data/Fig3D_STING_highlighted.pdf]

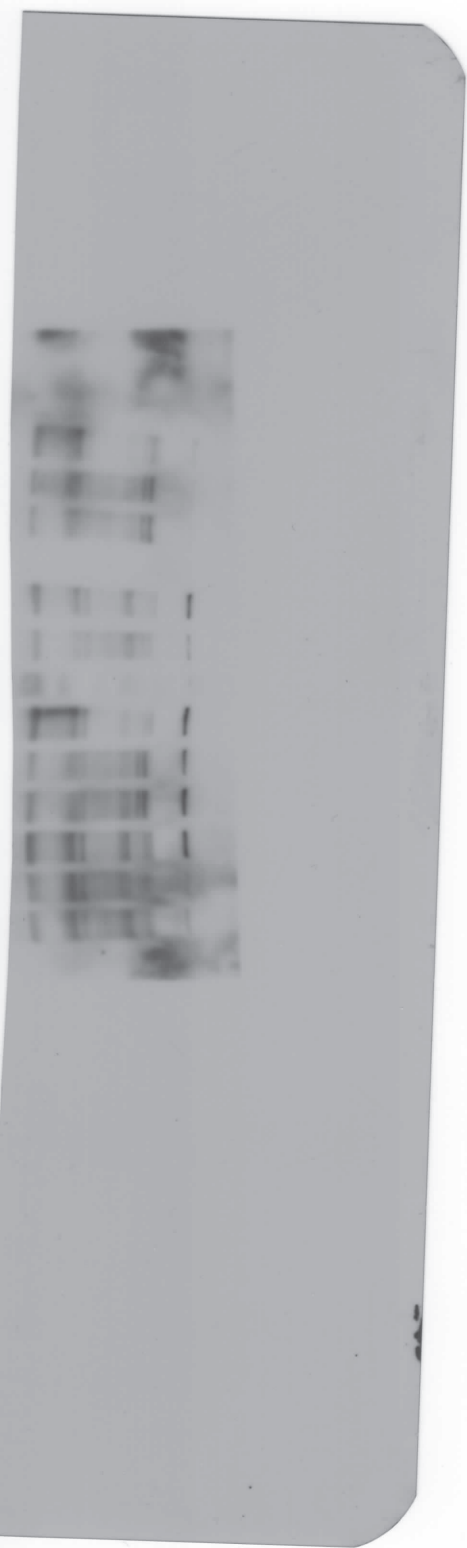

Supplement: Source data 1. [file elife-70436-supp2.zip › Western Blots Source Data/Fig6A_STING_uncropped.pdf]

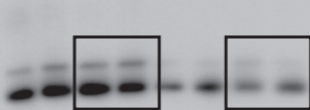

Supplement: Source data 1. [file elife-70436-supp2.zip › Western Blots Source Data/Fig6_S1B_mcherry_highlighted.pdf]

(1) → puffer LPS  
(2) → winter LPS  
(3) → invirogan LPS ] for

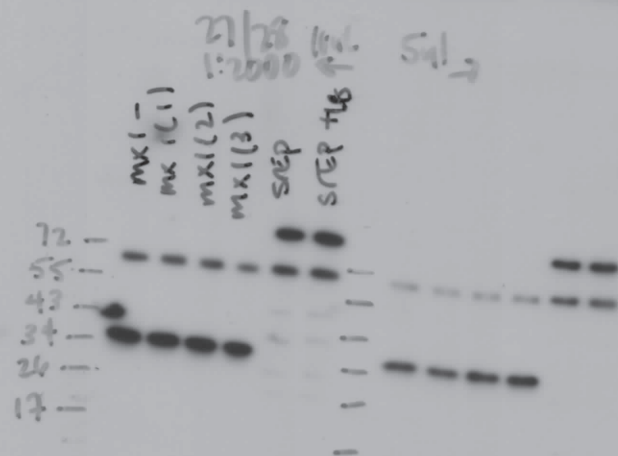

Supplement: Source data 1. [file elife-70436-supp2.zip › Western Blots Source Data/Fig2_S1C_STING_uncropped.pdf]

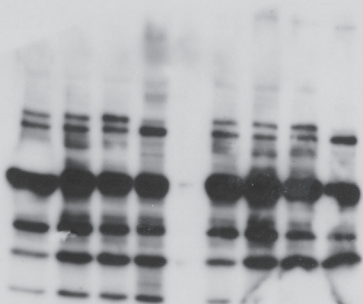

Supplement: Source data 1. [file elife-70436-supp2.zip › Western Blots Source Data/Fig3E_STING.pdf]
